# Comparative analysis of spatiotemporal gait parameters in patients with distal femoral megaprosthesis and healthy subjects using an inertial measurement unit (IMU)

**DOI:** 10.1017/wtc.2025.10009

**Published:** 2025-06-13

**Authors:** Nadia Jover-Jorge, Paula González-Rojo, José Vicente Amaya-Valero, Francisco Baixauli-García, Carolina de la Calva-Ceinós, Manuel Angulo-Sánchez, Javier Martínez-Gramage, Juan Francisco Lisón

**Affiliations:** 1 Department of Orthopedic Surgery and Traumatology, Hospital Universitario y Politécnico la Fe, Valencia, Spain; 2 Department of Biomedical Sciences, Universidad Cardenal Herrera-CEU, CEU Universities, Valencia, Spain; 3 Centre of Physiopathology of Obesity and Nutrition (CIBERobn), Instituto de Salud Carlos III, Madrid, Spain; 4 Orthopedic Surgery and Traumatology Research Group, Instituto de Investigación sanitaria la Fe, Valencia, Spain; 5 Department of Physiotherapy, Universidad Cardenal Herrera-CEU, CEU Universities, Valencia, Spain

**Keywords:** Megaprosthesis, Limb salvage surgery, Gait analysis, Inertial measurement unit (IMU), Spatiotemporal gait parameters, Distal femur tumour

## Abstract

Limb salvage surgery (LSS) with megaprosthesis is a common treatment for distal femur tumors, but its impact on gait remains poorly understood. Traditional gait analysis methods are costly and require specialized equipment. This study aims to compare spatiotemporal gait parameters between patients with distal femur megaprosthesis and healthy controls using an inertial measurement unit (IMU). We conducted a case–control study with 79 participants: 31 patients with distal femur megaprosthesis and 48 healthy controls. Gait data were collected using an IMU placed at L5-S1, capturing metrics such as gait quality index (GQI), pelvic kinematics, propulsion index, and gait speed. Statistical analysis included Student’s *t*-test, Mann–Whitney U test, and one-way ANOVA to compare gait parameters across groups. Patients with megaprosthesis exhibited significantly lower gait speed, propulsion index and anteroposterior acceleration symmetry index compared to controls (*p* < .05). GQI was reduced in the healthy legs of the cases (92.3%) compared to control legs (96.6%). Adaptations included prolonged stance phases in healthy legs and decreased single support phases in prosthetic legs. Despite these changes, gait patterns remained within functional ranges. IMU-based gait analysis reveals significant but functional alterations in gait mechanics among patients with distal femoral megaprosthesis. These findings underscore the need for tailored rehabilitation strategies to address compensatory mechanisms, optimize mobility, and enhance long-term outcomes. The use of IMU technology offers a cost-effective and portable alternative for clinical gait assessments.

## 1. Introduction

Limb salvage surgery (LSS) in tumor pathology is the treatment of choice for musculoskeletal tumors (Ilyas et al., 2001; Smolle et al., 2019). This approach aims to preserve limb function while ensuring oncological safety, but often results in altered gait mechanics due to the presence of a megaprosthesis.

The enhanced survival rates of these patients, attributed to progress in medical care, surgical methods, and the creation of megaprosthesis, have sparked a rising interest in the functional results and biomechanical effects of these individuals (Enneking, 1993; Gosheger et al., 2006; Carty et al., 2009b; Bekkering et al., 2012a, 2012b; Wilson et al., 2019; Jover-Jorge et al., 2024). However, there are limited data in the literature that objectively explain how the placement of these prostheses affects the gait parameters of patients (De Visser et al., 2000; De Visser et al., 2003; Carty et al., 2009a; Okita et al., 2013; Nakamura et al., 2014; Bruns et al., 2016; Morri et al., 2018).

Three-dimensional motion analysis systems that use cameras can provide accurate and impartial measurements (Park et al., 2005); however, these systems typically require patients to visit specialized facilities for motion analysis. The equipment is costly, lacks portability, and demands a high level of technical expertise along with labor-intensive calibration, which limits its use in routine clinical environments. Often, clinicians do not have access to objective biomechanical data needed to assess patient performance. As a result, a more comprehensive and practical strategy is necessary to quantify clinical settings. A promising solution to this challenge is the use of inertial measurement units (IMUs), which offer objective assessments of movement patterns during practical tasks in healthcare settings. IMU motion capture technologies are lightweight, less expensive than traditional camera systems, and are increasingly being used for gait analysis and mobility assessments in orthopedics and sports medicine (Zecca et al., 2013; Al-Amri et al., 2018; Teufl et al., 2019; Martínez-Gramage et al., 2020; Moltó et al., 2020; Sharifi Renani et al., 2020; Latajka et al., 2023).

These devices can be attached to a body part to determine its movement in a three-dimensional space. IMU-based gait analysis has the capability to facilitate quick setup and data collection across various gait protocols and settings.

Our assumption was that notable variations would be detected in gait parameters, highlighting the effect of surgical treatment on mobility and general functionality. The purpose of our research was to analyze spatiotemporal gait parameters between individuals who received megaprosthesis reconstruction after LSS for a malignant tumor in the distal femur and healthy individuals, using an IMU. We analyze the differences between cases and controls in anteroposterior acceleration symmetry index, pelvic symmetry, and range of motion parameters. To evaluate differences in the gait quality index, propulsion and gait cycle phases (stance phase, swing phase, first double support phase, and single support phase), we considered three groups: control leg (the mean values of the right and left legs of the controls), megaprosthesis leg (MP, pathological leg with prosthesis in the cases), and healthy leg (HL, unaffected leg in the cases).

## 2. Methods

### 2.1. Study design

Study design was a single-center case–control study and included a comparative analysis of gait patterns, focusing on key metrics such as gait quality index, anteroposterior acceleration symmetry index, 3D pelvis kinematics, and gait speed (ClinicalTrials.gov NTC 05202873). The research protocol received endorsement from the Human Ethics Committee of Hospital La Fe (2017/0336) and the Ethics Committee for Biomedical Research of the CEU Cardenal Herrera University (CEI21/062). All methodologies were executed in alignment with the tenets established in the World Medical Association’s Declaration of Helsinki, and informed written consent was secured from all participants before their enrolment in the research.

We undertook a comprehensive retrospective analysis aimed at identifying all occurrences of distal femoral megaprosthesis implanted between January 2013 and February 2024, as recorded in our tumor registry. A cumulative total of 42 patients were deemed eligible for inclusion in the study. Of these, five individuals had unfortunately succumbed. The remaining 37 patients were subsequently contacted through telephone communication. Among this cohort, four individuals opted out of participation, and two experienced considerable challenges with ambulation, culminating in a final participant group of 31 patients. The overall rate of compliance for participation was recorded at 73.8%.

### 2.2. Eligibility criteria

The inclusion criteria for the case group were made up of individuals over 16 years old who had surgery involving a distal femur megaprosthesis for tumor-related reasons at Hospital La Fe. These individuals were treated with either a traditional cemented or cementless system, or a biological osteointegration system (Compress®), and minimum postoperative follow-up period of 3 months.

The control group was made up of volunteer patients aged 16 and older who did not have joint prostheses in their lower limbs or any prior musculoskeletal or neurological conditions that might affect their gait.

Exclusion criteria included individuals unable to walk at the time of the study (due to medical or musculoskeletal issues), patients who were terminally ill and receiving palliative care, or those with prior neurological or musculoskeletal conditions that could impede their ability to walk.

### 2.3. Data collection

Various demographic factors were gathered, including age, body mass index (BMI), time since surgery (in months), percentage of femur remaining healthy after tumor resection (% bone remnant), and leg length discrepancy (in millimeters).

**Figure 1.**
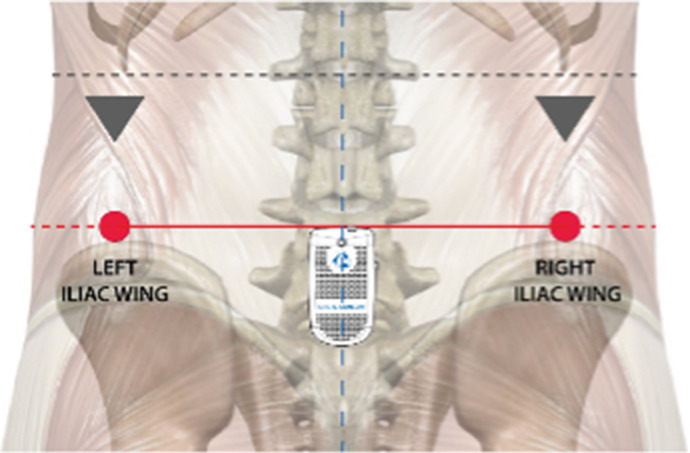
Anatomical references for the placement of the IMU (Bioengineering, 2016).

Gait assessment was performed using an IMU BTS G-Sensor (BTS Bioengineering, Garbagnate Milanese, Italy) with an ergonomic belt at the height of L5-S1 (Figure 1).

The device automatically calibrates itself whenever the walking test function is used for a specific patient. This IMU comprised a 16-bit triaxial accelerometer with multiple sensitivities (± 2, ± 4, ± 6, ± 8, and ± 16 g) with a frequency of 4–1,000 Hz, a triaxial gyroscope with multiple sensitivities (± 250, ± 500, ± 1,000, ± 2,000 o/s), with a frequency oscillating between 4 and 8,000 Hz, and a triaxial 13-bit magnetometer (± 1,200 uT), with a frequency exceeding 100 Hz. The sensor has been validated in previous studies (Al-Amri et al., 2018; De Ridder et al., 2019; Teufl et al., 2019; Martínez-Gramage et al., 2020; Moltó et al., 2020; Sharifi Renani et al., 2020; Latajka et al., 2023).

### 2.4. Outcome measures

We conducted an assessment based in previous gait studies in people with malignant tumor (Latajka et al., 2023). Participants completed the Walk+ test, consisting of walking a 7-m hallway six times (three round trips), with turns at each end. Only straight-line walking segments were used for the analysis. This protocol allowed the collection of a sufficient number of gait cycles for reliable analysis, while maintaining a controlled assessment environment by avoiding variations in speed and surface incline. Raw triaxial accelerometer and gyroscope data were recorded at 100 Hz using the BTS G-Walk® sensor (BTS Bioengineering, Italy), positioned at the L5-S1 spinal level. Data were processed with BTS G- Studio® software (v2.8.16.1), which applies automatic gait cycle segmentation and parameter extraction according to the validated Walk+ protocol. A second-order low-pass Butterworth filter with a 10 Hz cutoff frequency was applied to minimize high-frequency noise. Gait cycles were segmented using vertical acceleration peaks to identify heel strikes, based on adaptive thresholding methods validated in previous IMU-based gait studies. The software computed step-by-step metrics from each cycle. For each participant, the reported value for each parameter corresponds to the mean of all valid steps during straight-line walking. Gait analysis parameters include:

- Gait Quality Index: incorporates the stance-swing phase ratio, reflecting temporal balance during walking (Faisal et al., 2024)
- Propulsion Index: measures the subject’s ability to propel the center of mass forward during the single support stance phase. Lower values may indicate impaired strength or propulsion asymmetry (Bernardini et al., 2019).
- 3D Pelvic Kinematics: includes the range and symmetry index of tilt, obliquity, and rotation, derived from angular velocity data, allowing assessment of trunk control and limb coordination (Queen et al., 2020; Bergamini et al., 2022).
- Anteroposterior acceleration symmetry index: quantifies the symmetry of forward acceleration between limbs, calculated using normalized bilateral differences (Macellari et al., 1999).
- Gait speed: estimated using anthropometrically scaled stride length and step frequency, voiding double integration of acceleration and minimizing drift via sensor fusion algorithms embedded in the IMU.
- Stance Phase: portion of the gait cycle during which the foot is in contact with the ground (~60%), including heel strike, midstance, and toe-off (Perry and Burnfield, 2010).
- Swing Phase: period when the foot is in the air (~40%), comprising acceleration, midswing, and deceleration (Whittle, 2014).
- Double Support Phase: when both feet are simultaneously in contact with the ground, ensuring balance during step transition (Gage et al., 2009).
- Single Support Phase: period when only one foot supports the body, indicating dynamic stability (Sutherland and Davids, 1993). Although only one IMU was used, the software distinguishes left and right limb contributions based on pelvic rotation and lateral acceleration patterns. This allows for estimation of limb-specific parameters and symmetry indices. The lumbar sensor placement also enables simultaneous for pelvic kinematics and overall gait dynamics with minimal patient burden.

### 2.5. Statistical analysis

No prospective data are available comparing gait parameters assessed using Inertial Measurement Units between patients with distal femur tumor prostheses (case group) and a control group of healthy volunteers (control group). Thus, we hypothesized that patients with distal femur tumor prostheses would have worse outcomes compared to the control group across all variables. We performed a power analysis using G*Power software v3.1.9.2 (Heinrich-Heine-University, Düsseldorf, Germany) and determined that a sample size of 68 participants would provide 85% statistical power at a 5% significance level (two-sided test) for a medium effect size (*d* = 0.7). The sample size was increased by 15% to account for potential dropouts, resulting in a final sample size of 78 participants. The assumption of normality for each dependent variable and study group was evaluated using the Kolmogorov–Smirnov test. Differences between the case group (patients with distal femur tumor prostheses) and the control group (healthy volunteers) across the various dependent variables were analyzed using either the Student’s *t*-test for independent samples (anteroposterior acceleration symmetry index, tilt symmetry index, tilt range, obliquity range, rotation range, and gait speed) or the Mann–Whitney U test (obliquity symmetry index and rotation symmetry index), based on whether the normality assumption was satisfied. To calculate the intrasubject variability, we computed the standard deviation of the anteroposterior acceleration symmetry index for each participant. To assess differences in variability between cases and controls, we used the Mann–Whitney U test. To compare the differences among the three groups, the control leg (mean values of the right and left leg of each control), the megaprosthesis leg (MP, tumor-affected leg treated with surgery in the cases), and the healthy leg (HL, healthy leg of the cases), in the gait quality index, stance phase, swing phase, first double support phase, single support phase, and propulsion, one- way ANOVA tests followed by Bonferroni post hoc tests were performed. All analyses were conducted using SPSS version 29.0.2.0 for Mac (SPSS, Inc., Chicago, IL, USA). A *p-*value of <.05 was considered the threshold for statistical significance in all comparisons.

## 3. Results

This study included 79 participants: 31 individuals in the case group (patients with distal femur tumor prostheses) and 48 healthy individuals in the control group. Table 1 summarizes the demographic and clinical characteristics of the participants.

**Table 1.** Demographic and clinical characteristics of the study population

	Group	
	Control (*n* = 48)	Cases (*n* = 31)
Age	42.4 (13.8)	39.3 (15.7)
Men	17	17
Women	31	14
BMI	23.3 (2.9)	24.9 (4.5)
Evolution (months)	-	44.2 (42.3)
Bone remnant (%)	-	63.5 (10.3)
Leg length discrepancy (mm)	-	16.4 (16.2)

*Note.* Values are presented as mean (standard deviation).

The primary reasons for endoprosthetic replacement among the case group included osteosarcoma (20 patients), chondrosarcoma (3 patients), giant cell tumor (4 patients), metastasis (2 patients), plasmacytoma (1 patient), and undifferentiated soft tissue sarcoma affecting bone (1 patient). Of the 31 patients, 18 had a single surgery to implant the prosthesis prior to gait analysis. Eight patients required a second surgery: four due to aseptic loosening of the prosthesis, two because of tumor recurrence, one due to prosthetic infection, and one after a pathological fracture related to a giant cell tumor, which had previously been treated with curettage and filling. Three patients needed a third surgery, with two undergoing further intervention due to infection in the previous prosthesis and one for recurrent tumor growth. Ultimately, two patients required a fourth surgery, both for aseptic loosening of the previous prosthesis.

No significant differences in age, gender, or BMI were observed between the case and control groups (*p* > .05). Following limb-sparing surgery, the case group showed an average residual bone length of 63.5% (± 10.3) and a leg length discrepancy averaging 16.4 mm (± 16.2).

The anteroposterior acceleration symmetry index, tilt symmetry index, obliquity range and gait speed were significantly lower in cases compared to controls, while no differences were observed in tilt range and rotation range (Table 2).

**Table 2.** Results of the independent *t*-tests comparing the case and control groups

	Group		Difference (95% CI)	*p*
	Control (*n* = 48)	Cases (*n* = 31)		
Anteroposterior acceleration symmetry index (%)	95.4 (3.9)	86.6 (7.0)	8.7 (5.7 to 11.8)	<.001**
Tilt symmetry index (%)	62.9 (27.7)	32.7 (24.7)	30.2 (17.1 to 43.2)	<.001**
Tilt range (°)	6.3 (2.4)	5.7 (2.2)	0.6 (−0.6 to 1.8)	.313
Obliquity range (°)	10.2 (0.4)	7.6 (0.8)	2.6 (1.0 to 4.2)	.002*
Rotation range (°)	10.4 (0.6)	10.8 (1.0)	−0.4 (−2.6 to 1.9)	.747
Gait speed (m/s)	1.48 (0.16)	1.25 (0.3)	0.23 (0.08 to 0.38)	.003*

*Note.* Values are presented as mean (standard deviation). ** p<0,01 and *p<0,05

The Mann–Whitney U test results showed higher values in the control group compared to the case group for the obliquity symmetry index and the rotation symmetry index (Table 3).We found statistically significant differences in the standard deviation of the anteroposterior acceleration symmetry index between cases and controls, with greater variability observed in cases compared to controls (*p* = .007). The ANOVA results revealed statistically significant between-group differences in the gait quality index, stance phase, swing phase, first double support phase, single support phase, and propulsion index. Bonferroni pairwise comparisons are presented in Table 4. Changes in gait parameters represent significant differences in the gait quality index (GQI) of the HL compared to controls (*p* < .001), being 96.6% in controls and 92.3% in the HL of the cases (Table 4). Specifically, regarding the stance and swing phases, patients with tumors modify the gait cycle of their HL, significantly increasing the stance phase and decreasing the swing phase, while the MP leg maintains the gait cycle like the legs of the controls (Table 4).

**Table 3.** Results of Mann–Whitney U test

	Median		IQR		Z	*p*
	Control	Cases	Control	Cases		
Obliquity symmetry index (%)	98.4	88.8	0.9	19.1	−5.350	<.001**
Rotation symmetry index (%)	95.5	93.8	6.5	7.4	−2.070	.038*

Abbreviations: IQR, Interquartile Range; Z, Mann–Whitney Z-statistic; **p < .001; *p < .05.

**Table 4.** Results of ANOVA

Group			Control minus MP leg		Health leg minus control		Health leg minus MP leg	
Control	MP leg	Health leg	Difference (95% CI)	*p*	Difference (95% CI)	*p*	Difference (95% CI)	*p*
**Galt quality index**								
96.6 (2.3)	93.9 (4.5)	92.3 (7.1)	2.7 (−0.1 to 5.5)	0.056	−4.3 (−7.0 to −1.5)	< 0.001**	−1.6 (−4.7 to 1.6)	0.673
**Stance phase**								
59.4 (1.6)	59.3 (3.8)	62.8 (4.5)	0.2 (−1.7 to 2.1)	1.000	3.3 (1.4–5.2)	< 0.001***	3.5 (1.45.7)	< 0.001***
**Swing phase**								
40.6 (1.6)	41.0 (4.0)	37.2 (4.5)	−4.2 (−2.4 to 1.5)	1.000	−3.3 (−5.3 to −1.4)	< 0.001**	−3.8 (−6.0 to −1.5)	< 0.001**
**First double support**								
9.6 (1.6)	10.6 (2.6)	11.3 (3.2)	−1.1 (−2.4 to 0.4)	0.230	1.7 (0.2–3.1)	0.016*	0.6 (−1 to 2.3)	1.000
**Single support**								
40.4 (1.7)	37.3 (4.5)	40.7 (3.9)	3.0 (1.1–5.0)	< 0.000**	0.4 (−1.6 to 2.3)	1.000	3.4 (1.25.6)	0.001*
**Propulsion index**								
93.3 (2.1)	7.7 (3.0)	7.4 (2.9)	1.7 (0.1–3.2)	0.030*	−1.9 (−3.4 to −0.4)	0.008**	−0.3 (−2.0 to 1.4)	1.000

*Note.* Values are presented as mean (standard deviation). *p-*values obtained from one-way analysis of variance (Bonferroni post hoc test).** p<0,01 and *p<0,05

Abbreviations: CI, confidence interval; MP, megaprosthesis.

Patients with tumors increase the first double support phase with their HL compared to this phase in controls. Additionally, they decrease the single support phase of their MP leg compared to the controls and their HL (Table 4).

Statistically significant differences were observed in the propulsion index, with decreases in both the MP leg and the HL of the cases compared to the legs of the controls (Table 4).

## 4. Discussion

Gait analysis plays a significant role in evaluating and managing patients with tumoral prosthesis or megaprosthesis. It provides objective data on gait patterns, which is crucial for assessing functional outcomes and guiding rehabilitation strategies (Jover-Jorge et al., 2024).

Studies have shown that patients with megaprosthesis exhibit gait abnormalities, but the evaluation methods and the variables studied are very heterogeneous, which makes it difficult to draw generalizable conclusions (Pesenti et al., 2018; Pellegrino et al., 2020). These abnormalities are often due to muscle weakness and joint stiffness resulting from the extensive surgical procedures required for tumor resection and prosthetic implantation (Jover-Jorge et al., 2024).

Gait analysis can help identify specific compensatory mechanisms used by patients, such as increased reliance on the contralateral limb and nonoperated joints, which may lead to secondary musculoskeletal impairments (Okita et al., 2013). This information is valuable for tailoring rehabilitation programs to address these compensations and improve overall gait efficiency.

The GQI quantifies the degree of deviation from the average gait of a control population, considering key parameters such as temporal–spatial, kinematic, and kinetic factors. It also assesses the subject’s ability to distribute the phases of the gait cycle – specifically the stance and swing phases – symmetrically and efficiently between the right and left sides. A perfect score of 100 is achieved when the stance phase accounts for exactly 60%, and the swing phase accounts for 40% of the total gait cycle, indicating optimal balance and coordination (Kingsbury et al., 2017). However, we acknowledge that these values may oversimplify natural gait variability, as the stance in healthy individuals generally ranges from 58 to 62% at comfortable walking speeds and tends to decrease as speed increases. Therefore, the 60/40 ratio should be understood as a normative reference rather than a rigid cutoff, and the GQI interpreted within the context of gait speed and individual variability. In our study, we observed a significant difference between the cases and controls regarding the GQI, with the HL group of the case group showing a value of 92.3% compared to 96.6% in the control group. According to Kingsbury et al., 2017, gait values above 90% are considered functional. Therefore, despite some asymmetry, the gait of patients with megaprosthesis in our study falls within a functional gait range. Future work may explore the use of speed-adjusted thresholds to refine the interpretation of this index.

De Visser et al., 2000 found results similar to ours, the MP leg shows a shorter stance phase than the HL leg. However, we did not find statistically significant differences between the stance phase of the MP legs with the control group. These findings suggest that the unaffected limb adopts a compensatory biomechanical strategy to optimize the function of the megaprosthesis (MP) during gait. Specifically, the reduced swing phase duration (37.2%) and the increased stance phase led to an elevated initial double support phase (11.3%). This adaptation likely enhances stability and facilitates the progression of the prosthetic limb, highlighting the role of interlimb coordination in accommodating functional asymmetries.

According to Pesenti et al., 2018, patients with megaprosthesis exhibit decreased knee flexion throughout the stance phase, which is attributed to quadriceps weakness. This reduced knee flexion can impact the overall gait pattern and may contribute to concerns about prosthesis wear over time. This loss of strength and range of motion may be due to the type of surgery performed. Understanding the implications of these surgical techniques is crucial for developing targeted rehabilitation strategies that can enhance functional outcomes and improve mobility in patients with megaprosthesis (Gu et al., 2024). Bernthal et al., 2015 also highlight that despite these alterations, patients with endoprosthetic reconstructions generally maintain an efficient gait and are active in their daily lives. This suggests that while the stance phase may be altered, functional outcomes can still be acceptable with appropriate rehabilitation and management. Jover-Jorge et al., 2024 also report good to excellent outcomes in 75% of patients, despite identifying significant differences in functional tests between cases and controls.

The literature does not reference the gait propulsion index (GPI) in patients with megaprosthesis. In our study, we observed a lower GPI in these patients compared to controls. This reduction may be influenced by changes in the pelvic symmetry index, likely resulting from decreased pelvic mobility as indicated by a lower GQI. Addressing these factors should be a focus of rehabilitation strategies to improve gait mechanics and overall functionality.

Gait speed is a critical indicator of functional mobility and independence. Gait speed following knee endoprosthetic replacement varies between studies (De Visser et al., 2003; Colangeli et al., 2007; Carty et al., 2009a, 2009b), likely due to differences in experimental settings, patient age, and tumor treatment. In our study, the average walking speed of patients was 1.25 m/s (SD 0.3), closely matching that of healthy controls at 1.48 m/s (SD 0.16), with statistically significant differences between the two groups. This apparent similarity in gait speed despite underlaying biomechanical alterations is consistent with previous literature. For instance, McClelland et al., 2011 reported similar findings in patients after total knee arthroplasty, where gait speed was preserved but altered kinematics were evident. Previous studies report similar data on the walking speed of our patients (Cammisa et al., 1990; Kawai et al., 1999; Carty et al., 2009a), and find a significant decrease in gait speed of the patients. This reduction is influenced by several biomechanical factors including altered muscle function, joint kinematics, and the greater intrasubject variability in the anteroposterior acceleration symmetry index observed in the cases, indicating a less stable gait pattern, decreased mobility, and increased energy expenditure during ambulation (Gu et al., 2024). Understanding these factors is crucial for developing effective rehabilitation strategies that can enhance mobility and reduce the energy costs associated with walking.

Rompen et al., 2002 conducted gait analysis in a cohort including all femoral reconstructions and found a less pronounced deficit in velocity, with a 12% reduction in comparison to controls. De Visser et al. (2000), in a study including patients who underwent knee and hip surgeries, reported that patients achieved a preferred gait speed of 0.7 m/s compared to 1.1 m/s (SD 0.08) in controls.

This study has several limitations. Firstly, there is a possibility of selection bias, as patients who are satisfied with their surgical outcomes and exhibit greater ambulation ability may be more likely to participate in gait evaluations. Additionally, the variability in the extent of musculoskeletal resection required during surgical procedures, influenced by the tumor type and its extent, complicates comparisons among patients. Longitudinal analysis in musculoskeletal cancer patients also presents challenges due to mortality, complications, and different disease stages during follow-up. This factor must be considered, as it could bias the average outcomes. Moreover, comparing the operated limb with those of healthy controls may not adequately reflect patient-specific characteristics or pre-existing conditions that could influence outcomes, especially since activity levels and physical capacity prior to surgery were not considered. Finally, although the use of a 7-m hallway for repeated walking is consistent with real-world clinical settings, it may introduce slight variability due to turning maneuvers. We minimized this effect by analyzing only straight-line segments, following the Walk+ protocol, but acknowledge that continuous walking paths or treadmill-based protocols could further reduce this source of variability. Although our study was not designed to evaluate rehabilitation strategies, the observed gait alterations, particularly the reduced propulsion index and the asymmetric loading between the limbs, may inform future therapeutic approaches. Interventions aimed at improving hip and ankle force generation, enhancing pelvic stability, and addressing compensatory loading patterns through targeted strengthening, gait retraining, and sensorimotor exercises could be beneficial. These hypotheses should be explored and validated in future prospective interventional studies.

## 5. Conclusion

In conclusion, gait analysis is an invaluable tool for evaluating patients with megaprosthesis, offering insights into gait patterns and functional outcomes. Despite gait abnormalities observed in these patients, such as compensatory strategies and muscle weakness, functional gait is still achievable with appropriate rehabilitation. Our study demonstrated that patients with megaprosthesis exhibited gait within a functional range, with some deviations in gait phases that reflect adaptive strategies. These findings underscore the importance of targeted rehabilitation to address these compensations, optimize mobility, and improve long-term outcomes for these patients. Continued research is necessary to refine gait evaluation methods and further enhance rehabilitation strategies.

## Data Availability

Data can be made available to interested researchers upon request by email to the corresponding author.
